# Global research mapping of substance use disorder and treatment 1971–2017: implications for priority setting

**DOI:** 10.1186/s13011-019-0204-7

**Published:** 2019-05-17

**Authors:** Bach Xuan Tran, Mackenzie Moir, Carl A. Latkin, Brian J. Hall, Cuong Tat Nguyen, Giang Hai Ha, Nam Ba Nguyen, Cyrus S. H. Ho, Roger C. M. Ho

**Affiliations:** 1 Institute for Preventive Medicine and Public Health, Hanoi Medical University, Hanoi, Vietnam; 2 School of Public Health, University of Alberta, Alberta, Canada; 3 Institute for Global Health Innovations, Duy Tan University, Da Nang, Vietnam; 4 Center for Evidence-based Medicine, Nguyen Tat Thanh University, Ho Chi Minh City, Vietnam; 5 Center for Behavioral Medicine, Nguyen Tat Thanh University, Ho Chi Minh City, Vietnam; 6 Department of Psychological Medicine, National University Hospital, Singapore, Singapore; 7 Department of Psychological Medicine, Yong Loo Lin School of Medicine, National University of Singapore, Singapore, Singapore; 8 Bloomberg School of Public Health, Johns Hopkins University, Baltimore, MD USA; 9 Global and Community Mental Health Research Group, Faculty of Social Sciences, University of Macau, Macao, SAR People’s Republic of China

**Keywords:** Bibliometric analysis, Substance use disorder, Global, mapping, Substance abuse treatment, Alcohol, Smoking

## Abstract

**Background:**

Globally, substance use disorders are prevalent and remain an intractable public health problem for health care systems. This study aims to provide a global picture of substance use disorders research.

**Methods:**

The Web of Science platform was used to perform a cross-sectional analysis of scientific articles on substance use disorders and treatment. Characteristics of publication volume, impact, growth, authors, institutions, countries, and journals were examined using descriptive analysis and network visualization graphs.

**Results:**

Thirteen thousand six hundred eighty-five papers related to illicit drugs (5403), tobacco (4469), and alcohol (2137) use disorders and treatment were published between 1971 and 2017. The number of publications on Mindfulness and Digital medicine topics had the highest increase with more than 300% since 2003–2007 despite later presence than other methods. The number of papers on other non-pharmaceutical therapies (behavioral therapy, cognitive behavioral therapy, skills training or motivational interviewing) grew gradually, however, the growth rate was lower every 5-year period. The United States is the substance use disorder research hub of the world with the highest volume of publications (8232 or 60.2%) and total citations (252,935 or 65.2%), number of prolific authors (25 of top 30 or 83%) and institutions (24 of top 26 or 92%), formed the most international research partnerships (with 96 distinct countries). The international collaboration followed a pattern based on geographic proximity and cultural similarity**.**

**Conclusions:**

This study offers a comprehensive picture of the global trend of publications of substance use disorder. Findings suggest a need for research policy that supports the examination of interventions that culturally adhere to different local contexts to address substance use disorder in communities.

**Electronic supplementary material:**

The online version of this article (10.1186/s13011-019-0204-7) contains supplementary material, which is available to authorized users.

## Background

Substance use and misuse are globally prevalent and remain an ongoing health crisis affecting every region of the world. In 2016, the United Nations Office on Drugs and Crime (UNODC) estimated that 275 million people aged 15–64 used drugs at least once [1], and the prevalence of drug use and drug use disorders has increased significantly in the period 2010–2016 [2]. Likewise, global alcohol consumption rates per capita had slightly increased over a 15-year period [3]. Regarding tobacco smoking, in 2015, 20.2% of the world’s population aged > 15 years old were current smokers [4]. As a result, substance use disorders caused approximately 20 million disability-adjusted life years (DALYs) and 8.6 million years of life lost (YLL) across regions and countries [5–7]. The World Health Organization (WHO) estimated that in 2016, drug and alcohol use disorders were responsible for respectively 160,235 and 145,565 deaths, which increased markedly from 1990 [8, 9]. These ongoing burdens are becoming great challenges for health systems of every country [2].

In the past decades, treatments of substance use disorders, both non-pharmacological and pharmacological methods, have been well-documented [10]. In terms of tobacco use, along with nicotine replacement therapies, non-pharmacological treatments such as “counseling”, “self-help”, and “behavioral therapies” have been proven as effective therapies that can maintain smoking cessation more than 6-month follow up [11, 12]. Similarly, non-pharmacological approaches including “skills training”, “cognitive behavior therapy” and “family and couple therapy” are used widely to improve alcohol dependence [13]. For drug use disorder, substitutional treatments including methadone, buprenorphine or naltrexone maintenance treatment are used commonly as replacement therapies, along with traditional medicine and motivational enhancement therapies [14].

Prevention and management of substance use disorder have been considered one of the top priorities. Internationally, the WHO and the United Nations have been at the forefront with many efforts in synthesizing evidence and developing guidelines and frameworks to combat this public health crisis [15–18]. There has also been an increasing focus on developing a standard set of indicators, which allow to build an optimal monitoring system for harm reduction interventions [19]. Despite these efforts, gaps in research, training, treatment, service delivery, and capacity building related to substance use disorder are recognized, with greater negative impacts on low-to-middle income countries [20]. Moreover, these challenges vary across regions and countries significantly. This heterogeneity requires contextually sensitive approaches to the development and implementation of ‘locally’ compatible policy solutions. This study attempts to ‘take stock’ of the currently available substance use literature through the use of bibliometric methods [21]. In literature, few studies using this method to investigate the current status and tendency of research publications in substance misuse in Saudi Arabia [22] or drug/cocaine addiction only [23–25]. This work aims to provide clinicians, policymakers and other stakeholders a better understanding of 1) the trend and the current focus of international research efforts regarding substance use disorders; 2) what evidence is currently available on related subjects; and 3) where research, evidence and service gaps remain ongoing challenges.

## Methods

We used the Web of Science (WoS) to retrieve research publications focusing on substance use, comorbidity, treatment, and interventions. The WoS outweights other databases such as Scopus or MEDLINE. Initial, the WoS allows to extract information based on research disciplines that could not be done in other databases. Second, the WoS database comprises of leading and high impact scientific journals, while other databases included journals with a variety of quality [26–28]. Third, the WoS has a comprehensive coverage of scientific publications from 1900 until now with a diversity of research disciplines. Forth, the WoS has advantages in allowing to perform advanced search tool, refine the results to particular criteria, and evaluate the research productivity. This database offers a wide range of information about title, authors, keywords, sources/organizations, countries, languages, total citations as well as the average citation per literature. Our analysis focused on substance use disorder articles published from January 1st, 1971 to December 31st, 2017 in peer-reviewed journals. We did not include grey literature, conference proceedings, or books/book chapters in our analysis. Articles written in any language other than English were excluded.

### Search strategy

Our search strategy was performed according to following steps:

### Inclusion step

The literature from the WoS was retrieved using a set of search terms, focusing on 1) substance use disorder (including all illicit substances), 2) tobacco use, and 3) alcohol use disorder [29]. Synonyms for each search term were identified by research team including senior researchers (B.X.T and G.H.H) and junior researchers (C.T.N and N.B.N), whom had experiences in the field of substance use disorder. The keywords used were referred from previously published systematic reviews [30–33]. Our search query is outlined in Table 1.

**Table 1:** Search Query Text

First, we searched for the three main kinds of substance use disorder:	
(1) smoking OR tobacco-smoking OR nicotine OR tobacco-use-disorder* OR Cigar* OR Tobacco	
(2) substance-abuse OR substance-related-disorder* OR substance-abuse-intravenous OR drug-rehabilitation OR drug-usage OR drug-depend* OR substance-use-disorder* OR opioid-related-disorder* OR opioid-abuse OR opioid-addict* OR Drug-Abuse OR Drug-Addict* OR Marijuana-abuse OR Marijuana-addict*	
(3) alcohol*-drinking OR alcohol*-addiction OR alcohol*-abuse OR alcohol-rehabilitation OR alcohol-depend*	
Second, we developed separate search queries for several commonly used interventions and methods for treating substance abuse disorder. These included:	
(4) Behavioral therapy with six sub-fields**:** Cognitive behavioral therapy, self-help, Motivational enhancement therapy, Motivational interviewing, The Matrix Model, and 12 Step Facilitation Therapy,	
(5) Psychological treatment method with three sub-fields**:** Family therapy, Group counseling OR mixed counseling and Mindfulness,	
(6) Pharmacological therapy with four sub-fields**:** Nicotine replacement therapy and Non nicotine medication were for nicotine addiction, for alcohol abuse were Disulfiram therapy OR Naltrexone OR Campral, and for opioid addiction we applied: Alternative-Drug* OR Methadone OR Buprenorphine OR Naltrexone, and	
(7) Other treatment methods included**:** herbal medicine, digital medicine, telephony, and acupuncture.	
In final step, we connected query 1 through 3 with the “AND” operator with queries 4 through 7 [see Additional file 1].	

### Exclusion step

We excluded articles which were 1) published later than 31 December 2017; 2) documents that were not articles such as book chapters and conference proceedings; 3) without author details; and 4) written in any language other than English. We also used the WOS database functionality to exclude publications in unrelated fields of study [see Additional file 1].

### Data extraction

Data were exported from the WoS in text format and imported into Microsoft Excel for analysis. Exported data included: (1) Total number of publications by year for three types of substance use disorder (Illicit drugs, tobacco, and alcohol); (2) Name and details of journals; (3) Authors’ name, affiliation and number of publications; (4) Top cited articles; (5) Types of articles; (6) Title of the paper; (7) Year of publication; (8) Author’s and WoS’s keywords; (9) Number of citations of each article; and (10) Abstracts.

### Data analysis

Our analysis of authorship involved an initial sorting of data based on the number of authors, total number of citations, citations per paper, h-index, and how many papers were written collaboratively [34]. We directly downloaded these data via the WOS Citation report.

We used search terms (see Table 1 (1), (2), (3)) using Microsoft Excel to quantify the volume of publications related to (1) drugs use disorder; (2) nicotine dependency; and (3) alcohol addiction. We then applied search terms (see Table 1 (4), (5), (6)) to determine the number of publications focusing on the following therapies and interventions: (4) Behavioral therapy OR Behavioral treatment, (5) Psychological treatment method, (6) Pharmacological therapy OR Pharmacological-treatment, and (7) Other treatment methods.

Next, we tracked the growth of publications in different substance use disorders. We used the period 5-year intervals across 1998 to 2017 to evaluate the “index of change” for each intervention. This measure shows a change of a research field by comparing the growth of publication of one period compared with that of previous one [35].

VOSviewer software (http://www.vosviewer.com/) was used to 1) create visualization graphs indicating contributions and collaborative efforts of sixty-one countries with at least five publications; 2) Visualizing the co-occurrence terms in titles and abstracts of all publications with at least 250 times of presence [36]. Networks highlighted the trend and provide the insight of the development of substance use disorders in our dataset at any level: node, connection, network and overall system [37].

## Results

Table 2 illustrates general information of our dataset. This topic attracted the concern of research community that showed by the volume of publications increased markedly every 5 years, especially between 2013 and 2017 (one-third of all articles). Nearly 70% of the papers in all three methods was collaborative research of 2–3 authors and 4–6 authors, that is promised a main trend year after year, reflecting the multi-investigator in a research study [38]. Substance use disorder was the research field across disciplines, that was showed by half of the publication assigning to 2 or 3 research areas. The use of alcohol and tobacco is increasing rapidly in developing countries [39], however, most of the lead author are from developed countries (China was the only Asian country in the list of top 10 country of first author).

**Table 2:** General characteristics of selected articles

		Tobacco		Drug addiction		Alcohol	
Characteristic	Category	Number	Percent	Number	Percent	Number	Percent
Total number of papers		4469	100	5403	100	2137	100
Year of publication	2013–2017	1688	37.8	2090	38.7	850	39.8
	2008–2012	1302	29.1	1565	29.0	536	25.1
	2003–2007	803	18.0	855	15.8	364	17.0
	1998–2002	364	8.1	489	9.1	234	10.9
	1993–1997	198	4.4	278	5.1	126	5.9
	1988–1992	82	1.8	70	1.3	24	1.1
	< 1988	32	0.7	57	1.0	3	0.1
Number of authors	1	286	6.4	418	7.8	166	7.8
	2–3	1300	29.1	1691	31.3	730	34.2
	4–6	1772	39.7	2197	40.7	866	40.5
	7–10	903	20.2	905	16.7	309	14.5
	> 10	208	4.7	192	3.6	66	3.1
Number of subject category	1	2261	50.6	2492	46.1	970	45.4
	2	1709	38.2	2331	43.1	934	43.7
	3	470	10.5	557	10.3	220	10.3
	4	22	0.5	14	0.3	9	0.4
	5	4	0.1	8	0.1	3	0.1
Country of first author (top 10) (number of papers)	1	USA	2464	USA	3329	USA	1444
	2	England	321	Australia	208	Australia	80
	3	Canada	179	England	200	England	76
	4	Australia	167	Canada	195	Canada	63
	5	Netherlands	99	Italy	115	Netherlands	40
	6	Spain	90	Germany	82	Sweden	33
	7	China	64	Spain	80	Italy	27
	8	Italy	60	France	77	Germany	26
	9	Germany	57	Netherlands	70	Iran	20
	10	Switzerland	57	Switzerland	61	Switzerland	18

**Fig. 1:**
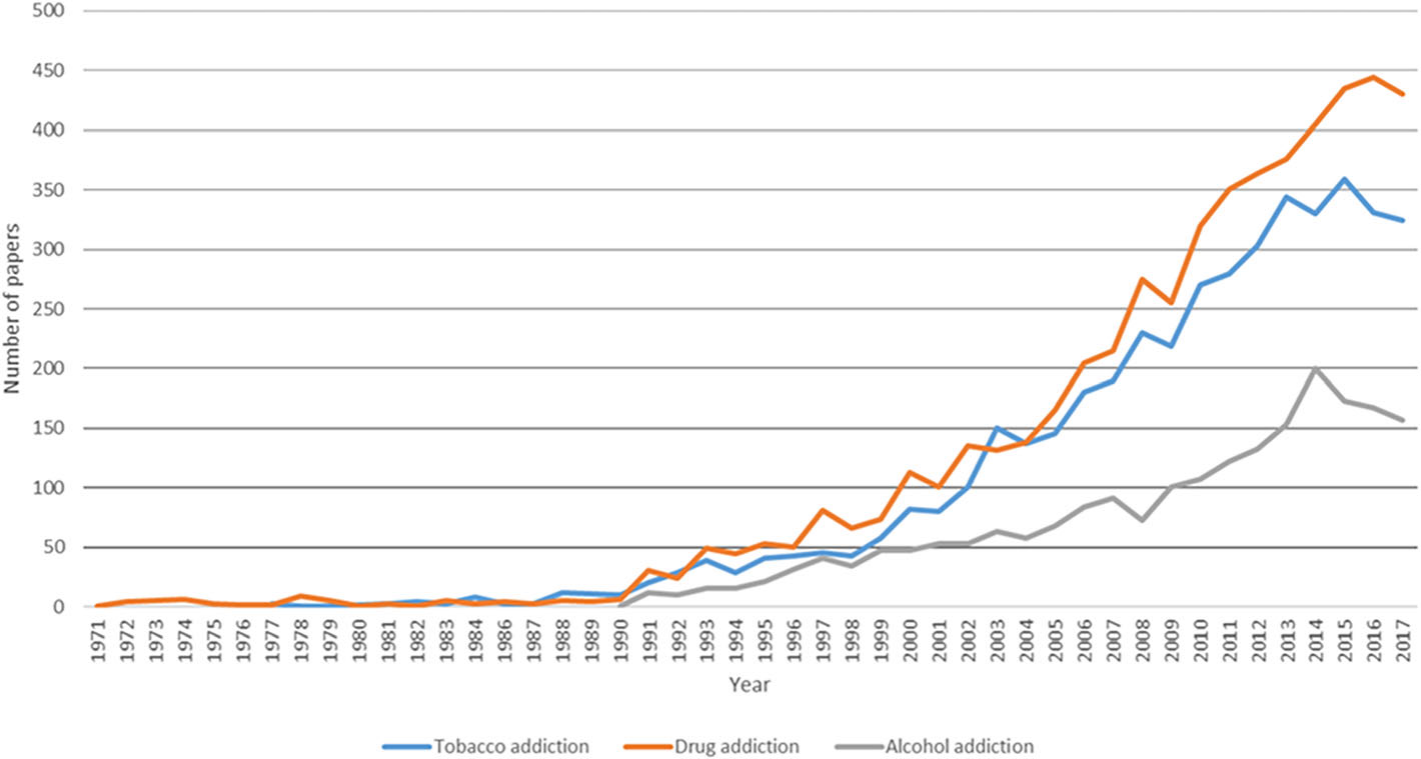
Number of papers by year in publication

Figure 1 reveals an acceleration of publications in the field of substance abuse disorder after 1990. The number of papers in this period accounted for approximately three-fourths of total number of publications. Noticably, the growth of publications was different among three types of substance use disorders **(**Table 3). The number of publications on Mindfulness and Digital medicine topics had the highest increase with more than 300% since 2003–2007 despite later presence than other methods. The number of papers on other non-pharmaceutical therapies (behavioral therapy, cognitive behavioral therapy, skills training or motivational interviewing) grew gradually, however, the growth rate was lower every 5-year period.

**Table 3:** Number of publication and the growth of publication (%)

	Number of paper (Growth of publication - %)														
	Tobacco					Drug					Alcohol				
Name of therapy	1993–1997	1998–2002	2003–2007	2008–2012	2013–2017	1993–1997	1998–2002	2003–2007	2008–2012	2013–2017	1993–1997	1998–2002	2003–2007	2008–2012	2013–2017
Non pharmaceutical therapy															
Behavioral therapy	2	5 (150)	19 (280)	44 (131.6)	72 (63.6)	84	187 (122.6)	326 (74.3)	620 (90.2)	876 (41.3)	47	106 (125.5)	157 (48.1)	242 (54.1)	453 (87.2)
Cognitive behavioral therapy	3	5 (66.7)	7 (40)	35 (400)	50 (42.9)	17	63 (270.6)	125 (98.4)	259 (107.2)	496 (91.5)	16	46 (187.5)	69 (50)	109 (58)	258 (136.7)
Skills training	7	4 (−42.9)	10 (150)	15 (50)	25 (66.7)	10	39 (290)	91 (133.3)	109 (19.8)	108 (−0.9)	11	40 (263.6)	56 (40)	78 (39.3)	80 (2.6)
Motivational interviewing	1	10 (900)	22 (120)	51 (131.8)	86 (68.6)	8	22 (175)	73 (231.8)	172 (135.6)	226 (31.4)	7	22 (214.3)	54 (145.5)	102 (88.9)	158 (54.9)
The Matrix Model	0	0 (−)	0 (−)	0 (−)	0 (−)	0	1 (−)	2 (100)	1 (−50)	1 (0)	0	0 (−)	0 (−)	1 (−)	1 (0)
12 Step Facilitation Therapy	0	0 (−)	1 (−)	0 (−100)	1 (−)	2	7 (250)	11 (57.1)	15 (36.4)	24 (60)	2	11 (450)	12 (9.1)	14 (16.7)	20 (42.9)
Psychological treatment	1	1 (0)	5 (400)	11 (120)	14 (27.3)	19	28 (47.4)	54 (92.9)	107 (98.1)	176 (64.5)	6	14 (133.3)	21 (50)	46 (119)	72 (56.5)
Family-therapy	2	3 (50)	1 (−66.7)	4 (300)	11 (175)	37	87 (135.1)	108 (24.1)	183 (69.4)	235 (28.4)	19	49 (157.9)	42 (−14.3)	63 (50)	73 (15.9)
Group counseling/ mixed counseling	30	57 (90)	113 (98.2)	189 (67.3)	219 (15.9)	16	35 (118.8)	49 (40)	93 (89.8)	123 (32.3)	7	13 (85.7)	27 (107.7)	30 (11.1)	54 (80)
Mindfulness	0	0 (−)	1 (−)	12 (1100)	52 (333.3)	0	1 (−)	6 (500)	38 (533.3)	115 (202.6)	0	1 (−)	4 (300)	17 (325)	70 (311.8)
Digital medicine	0	0 (−)	2 (−)	8 (300)	33 (312.5)	0	0 (−)	0 (−)	3 (−)	22 (633.3)	0	0 (−)	1 (−)	1 (0)	16 (1500)
Telephony	5	3 (−40)	10 (233.3)	18 (80)	27 (50)	0	4 (−)	12 (200)	25 (108.3)	32 (28)	1	2 (100)	9 (350)	11 (22.2)	34 (209.1)
Acupuncture	1	4 (300)	2 (−50)	10 (400)	15 (50)	4	9 (125)	3 (−66.7)	15 (400)	20 (33.3)	4	1 (−75)	4 (300)	8 (100)	11 (37.5)
Pharmaceutical therapy															
Nicotine replacement therapy	8	15 (87.5)	33 (120)	53 (60.6)	64 (20.8)										
Non-nicotine medication	1	2 (100)	1 (−50)	0 (−100)	0 (−)										
Herbal medicine	0	0 (−)	0 (−)	2 (−)	2 (0)										
Alternative-Drug						14	19 (35.7)	38 (100)	53 (39.5)	70 (32.1)					
Buprenorphine						15	36 (140)	107 (197.2)	223 (108.4)	328 (47.1)					
Naltrexone (for drug use disorder)						21	35 (66.7)	73 (108.6)	135 (84.9)	164 (21.5)					
Methadone						170	250 (47.1)	411 (64.4)	711 (73)	830 (16.7)					
Herbal medicine						0	1 (−)	6 (500)	5 (−16.7)	8 (60)					
Disulfiram therapy											4	8 (100)	12 (50)	10 (−16.7)	18 (80)
Naltrexone (for Alcohol)											15	35 (133.3)	43 (22.9)	60 (39.5)	77 (28.3)
Campral/acamprosate											0	6 (−)	5 (−16.7)	11 (120)	17 (54.5)
Herbal medicine											1	1 (0)	5 (400)	6 (20)	3 (−50)

Table 4 presents that the Journal of Substance Abuse and Treatment was the most common journal with the highest number of publications (613 papers), followed by the Drug and Alcohol Dependence (591) and the Addiction journal (400).

**Table 4:** Research area and the Journal included the research area

No	Research area	Total papers	Journal name	Total papers	Journal name	Total papers
1	Substance Abuse	4504	Journal of Substance Abuse Treatment	613	Addiction	400
			Drug and Alcohol Dependence	591	Addictive Behaviors	324
2	Psychiatry	3130	Drug and Alcohol Dependence	591	Psychopharmacology	197
			Addiction	400	Substance Use Misuse	162
3	Psychology	3041	Journal of Substance Abuse Treatment	613	American Journal of Drug and Alcohol Abuse	174
			Addictive Behaviors	324	journal of consulting and clinical psychology	172
4	Pharmacology pharmacy	1838	Journal of Substance Abuse Treatment	613	American Journal of Drug and Alcohol Abuse	174
			Addictive Behaviors	324	Journal of Consulting and Clinical Psychology	172
5	Public Environmental Occupational Health	1549	Nicotine Tobacco Research	316	BMC Public Health	85
			Preventive Medicine	92	American Journal of Preventive Medicine	65
6	General internal Medicine	1311	Cochrane Database of Systematic Reviews	152	American Journal of Preventive Medicine	65
			Preventive Medicine	92	JAMA Journal of The American Medical Association	56
7	Neurosciences Neurology	1195	Psychopharmacology	197	CNS Drugs	59
			Neuropsychopharmacology	74	Pharmacology Biochemistry and Behavior	57
8	Health Care Sciences Services	676	Journal of Medical Internet Research	91	Journal of General Internal Medicine	40
			Psychiatric Services	62	Journal of Behavioral Health Services Research	32

Table 5 shows information of 30 most prolific researchers. They published from 44 to 117 publications and had 1137 to 5878 citations. Among these authors, 25 (83%) had affiliations in the American institutions, while other authors worked at institutions in England, Italy and Canada.

**Table 5:** Most prolific authors

No	Author	Affiliation and country	Total papers	Total citations	Citations per paper	h-index	Papers in collaboration	Collaboration index (signatures per paper)
1	Kathleen M. Carroll	Yale University School of Medicine, United States	117	5878	50.2	41	114	6.4
2	Caryn Lerman	University of Pennsylvania, Abramson Cancer Center, Annenberg School for Communication, Department of Psychiatry, United States	87	4372	50.3	41	87	7.4
3	Nancy M. Petry	University of Connecticut Health Center, United States	74	2504	33.8	26	67	3.8
4	Robert West	St Georges University of London, England	72	3307	45.9	32	70	5.1
5	Frederick L. Altice	Yale University, Department of Internal Medicine, Infectious Diseases Section, United States	69	1913	27.7	27	69	6.3
6	Jon O. Ebbert	Department of Laboratory Medicine & Pathology, Mayo Clinic, United States	65	1253	19.3	20	64	4.9
7	Raymond Niaura	Brown University, United States	64	3276	51.2	33	63	7.8
8	Ling W	University of Pennsylvania School of Medicine, United States	63	2989	47.4	27	61	8.0
9	Maxine L. Stitzer	Johns Hopkins University School of Medicine, United States	63	3055	48.5	30	63	6.0
10	Jasjit S Ahluwalia	University of Kansas School of Medicine and Kansas Cancer Institute, United States	62	2045	33.0	28	62	7.2
11	Edward V. Nunes	Columbia University, United States	61	2034	33.3	25	61	8.9
12	Robert P. Schwartz	Social Research Center, Friends Research Institute, Baltimore, United States	55	1093	19.9	19	54	6.8
13	Timothy B. Baker	University of Wisconsin Medical School, United States	54	4080	75.6	30	54	8.7
14	David A. Fiellin	Division of Alcohol and Drug Abuse, McLean Hospital, United States	53	1949	36.8	26	53	7.6
15	Charla Nich	Yale University School of Medicine, United States	52	3223	62.0	28	52	6.9
16	Kevin E. O’grady	University of Maryland, United States	52	1072	20.6	18	52	6.6
17	Thomas R. Kosten	Yale University School of Medicine, United States	51	2059	40.4	25	51	5.7
18	Saul Shiffman	University of Tasmania, Australia	50	3201	64.0	31	46	3.8
19	Roger D. Weiss	McLean Hospital, United States	49	1151	23.5	20	48	7.8
20	Richard A. Brown	The Warren Alpert Medical School of Brown University, United States	47	1750	37.2	20	47	7.5
21	J. Taylor Hays	Nicotine Dependence Center, Mayo Clinic, United States	47	2404	51.2	21	47	5.7
22	Michael C. Fiore	University of Wisconsin Medical School, United States	46	3682	80.0	24	46	8.1
23	Icro Maremmani	Santa Chiara University Hospital, University of Pisa, Italy	46	636	13.8	12	46	7.0
24	Paul Aveyard	Psychology Department, King’s College London, England.	45	1463	32.5	23	45	6.4
25	Neal L. Benowitz	Brigham and Women’s Hospital, Boston, United States	45	2341	52.0	24	41	7.6
26	Bruce J. Rounsaville	Yale University School of Medicine, United States	45	3313	73.6	28	45	6.8
27	Ricard S. Schottenfeld	PT Foundation, United States	45	1428	31.7	21	45	7.2
28	John R. Hughes	Penn State University, United States	44	3293	74.8	25	32	3.3
29	Rudolf H. Moos	Department of Veterans Affairs, United States	44	2022	46.0	29	43	3.3
30	Rachel F. Tyndale	University of Toronto, Canada.	44	1137	25.8	17	44	7.8

Table 6 illustrates the research productivity among top 41 countries. The top five countries were North America (the United States of America and Canada), Europe (England and Italy), and Australia. The United States of America was the substance use disorder knowledge hub in the world, and ranked at the top of all indices and was the main collaborator of 38 countries in the list. There were 70% of research conducted in the U.S. as a result of national collaborations, whereas more than 40% of research projects in other countries were the results of international cooperation.

**Table 6:** Most prolific countries and the collaborations

Region	Rank	Countries	Total papers	%	Total citations	Citations per paper	Intra- country collaboration	%	Inter-country collaboration	%	Distinct countries of collaboration	Main collaborator (and number of collaborations)
North America	1	United States	8232	60.2	252,935	30.7	6403	77.8	1829	22.2	96	England (244)
	3	Canada	701	5.1	18,020	25.7	362	51.6	339	48.4	63	USA (216)
East Asia And Pacific	4	Australia	682	5	16,143	23.7	300	44.0	382	56.0	63	Wales (152)
	9	China	338	2.5	6720	19.9	94	27.8	244	72.2	55	USA (142)
	20	Japan	129	0.9	1991	15.4	87	67.4	42	32.6	47	USA (17)
	24	Taiwan	107	0.8	1597	14.9	65	60.7	42	39.3	26	USA (22)
	27	South Korea	100	0.7	1587	15.9	54	54	46	46	23	USA (31)
	28	New Zealand	99	0.7	2250	22.7	53	53.5	46	46.5	16	England (19)
	33	Hong Kong	68	0.5	1088	16	1	1.5	67	98.5	43	China (67)
	34	Malaysia	66	0.5	1005	15.2	17	25.8	49	74.2	42	USA (31)
	39	Singapore	42	0.3	1553	37	16	38.1	26	61.9	30	USA (10)
Europe and Central Asia	2	England	1064	7.8	37,226	35	510	47.9	554	52.1	71	USA (244)
	5	Italy	379	2.8	9989	26.4	190	50.1	189	49.9	54	USA (82)
	6	Netherlands	379	2.8	9771	25.8	185	48.8	194	51.2	53	USA (85)
	7	France	372	2.7	8250	22.2	150	40.3	222	59.7	49	USA (109)
	8	Germany	341	2.5	8503	24.9	148	43.4	193	56.6	46	USA (76)
	10	Spain	315	2.3	6734	21.4	178	56.5	137	43.5	66	USA (53)
	11	Switzerland	240	1.8	6077	25.3	70	29.2	170	70.8	52	USA (96)
	12	Sweden	232	1.7	7483	32.3	116	50	116	50	38	USA (37)
	14	Wales	197	1.4	6742	34.2	12	6.1	185	93.9	40	Australia (152)
	18	Norway	144	1.1	3992	27.7	67	46.5	77	53.5	31	USA (26)
	19	Denmark	132	1	4716	35.7	80	60.6	52	39.4	27	USA (19)
	21	Georgia	129	0.9	3044	23.6	3	2.3	126	97.7	16	USA (120)
	25	Scotland	101	0.7	3135	31	31	30.7	70	69.3	35	England (47)
	26	Finland	100	0.7	1981	19.8	47	47	53	53	28	Sweden (17)
	29	Belgium	96	0.7	2798	29.2	35	36.5	61	63.5	40	Netherlands (28)
	30	Turkey	93	0.7	967	10.4	79	84.9	14	15.1	24	Germany (3)
	31	Austria	78	0.6	1974	25.3	43	55.1	35	44.9	24	USA (19)
	32	Ireland	75	0.5	1373	18.3	52	69.3	23	30.7	33	England (10)
	35	Poland	63	0.5	1263	20.1	44	69.8	19	30.2	45	USA (10)
	38	Greece	42	0.3	1312	31.2	21	50	21	50	34	England (9)
	40	Portugal	39	0.3	1018	26.1	11	28.2	28	71.8	42	Italy (10)
Latin America and The Caribbean	17	Mexico	178	1.3	6888	38.7	16	9	162	91	43	USA (156)
	22	Brazil	127	0.9	2363	18.6	62	48.8	65	51.2	53	USA (30)
Middle East and North Africa	13	Israel	200	1.5	7132	35.7	65	32.5	135	67.5	46	USA (126)
	16	Iran	188	1.4	1396	7.4	98	52.1	90	47.9	39	USA (39)
	23	Oman	115	0.8	1901	16.5	1	0.9	114	99.1	48	USA (62)
	36	Lebanon	53	0.4	2713	51.2	1	1.9	52	98.1	34	USA (50)
	41	Jordan	34	0.2	252	7.4	4	11.8	30	88.2	11	USA (26)
South Asia	15	India	195	1.4	3887	19.9	71	36.4	124	63.6	43	USA (109)
Sub-Saharan Africa	37	South Africa	53	0.4	1166	22	25	47.2	28	52.8	27	USA (14)

**Fig. 2:**
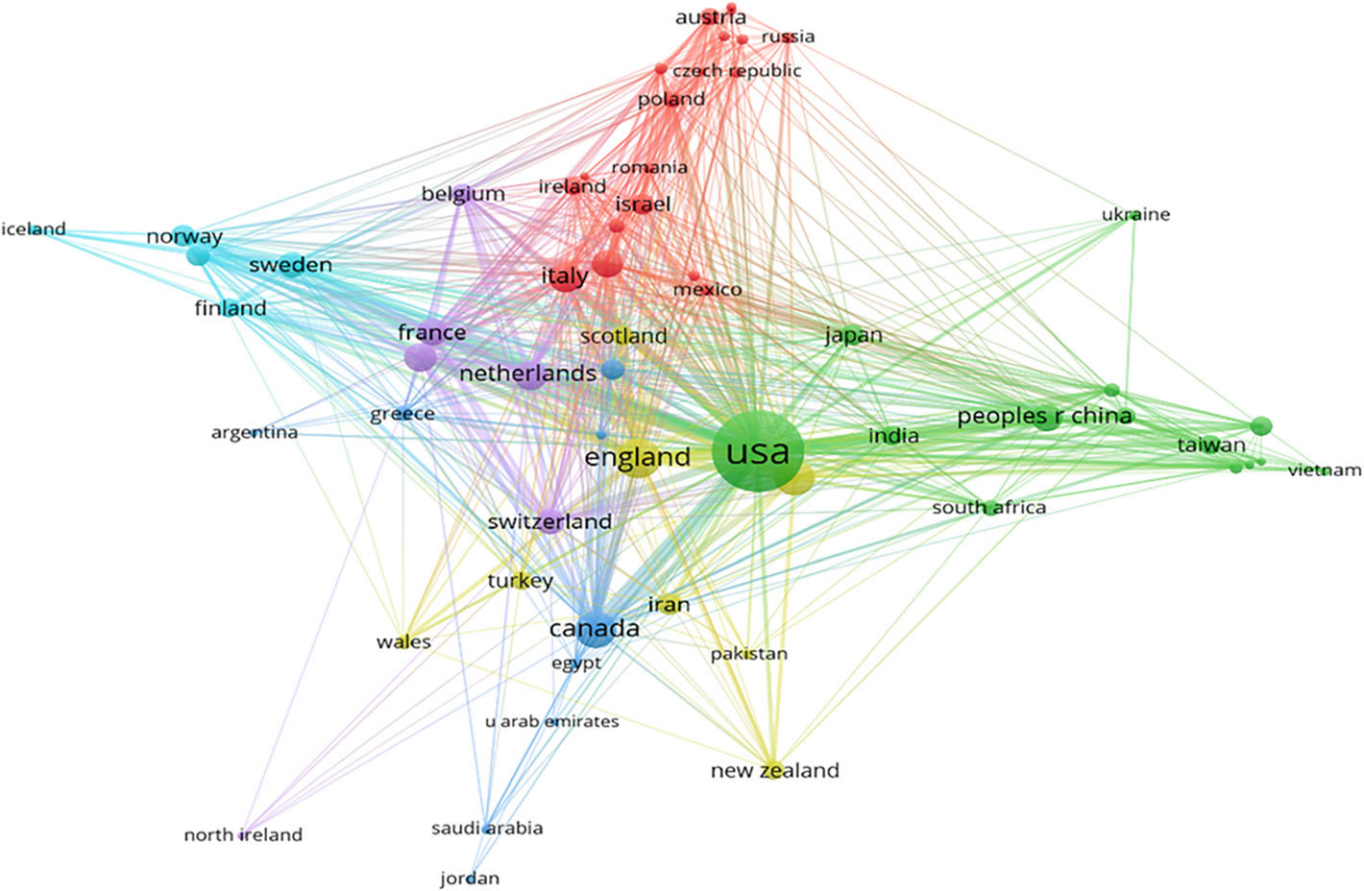
A global network of the 61 most prolific countries

Strength of collaborative partnerships and contributions among countries are shown in Fig. 2. There were three major geographical research clusters including: 1) England, Scotland, Wales and Turkey; 2) East, and South-East Asia, such as The Peoples Republic of China, India, Taiwan, and Vietnam; and 3) Central and Eastern Europe, for example, Austria, Poland, and Bulgaria. This distribution might be justified by the geographical proximity and cultural similarity among countries in each cluster.

**Fig. 3:**
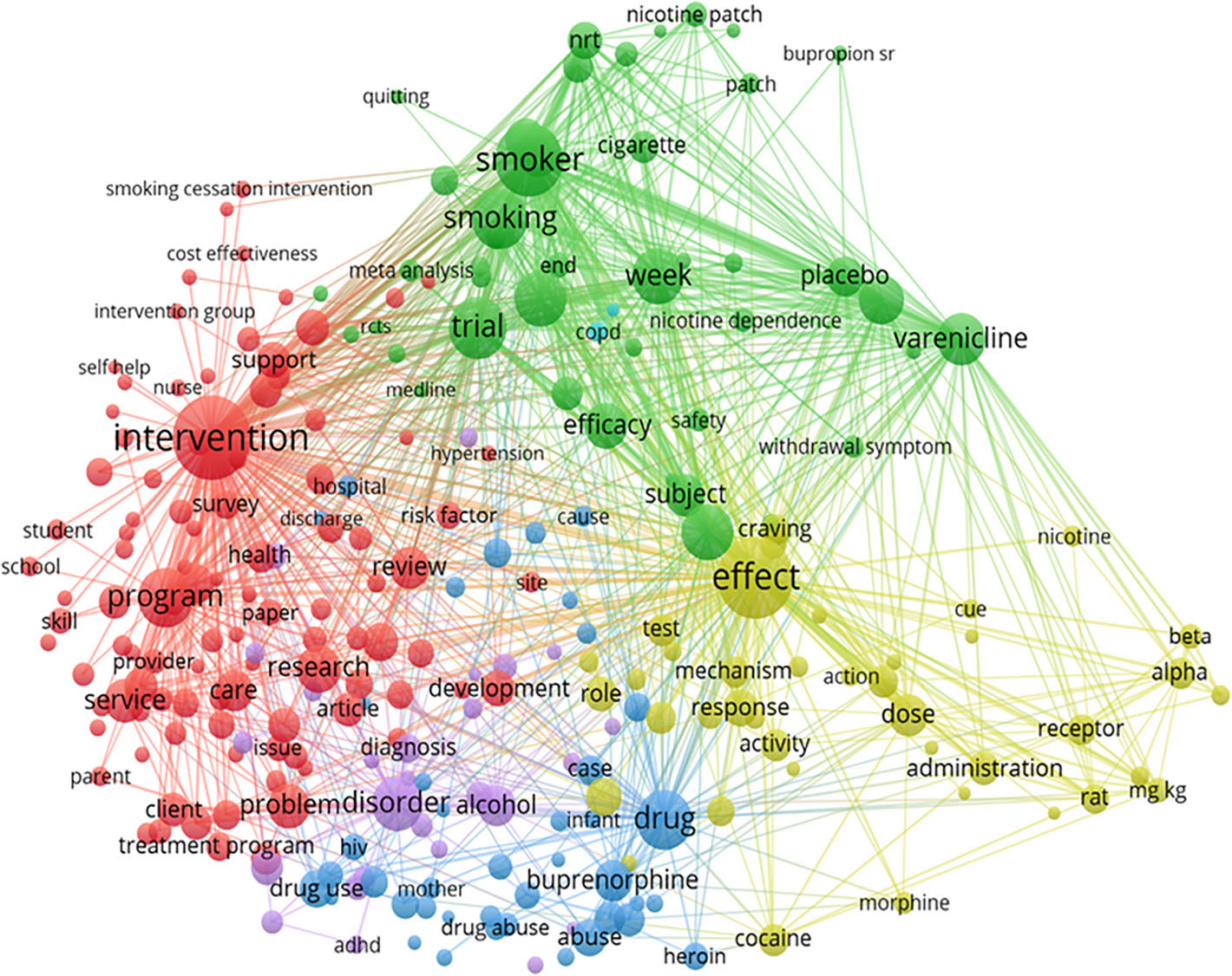
Text (Title and abstract) mining

Figure 3 presents the most popular terms with at least 250 appearances drawn from the title and abstract, which could be separated into three topic clusters: 1) Intervention (Red), 2) Types of addictions: Tobacco addiction (Green), Drug addiction (Blue), Alcohol (Purple), and 3) Effects (Yellow).

### Intervention

The word “Intervention” appeared the most with 9438 times appearances in papers focusing on smoking addition, followed by drug use and alcohol. Moreover, these papers presented intervention programs in different populations such as adolescents and students; and with a variety of methods such as training program, counseling, and motivational interviewing.

### Types of addiction

“Smoker” and “smoking” frequently appeared alongside “withdrawal”. The term “pharmacotherapy” appeared in papers focusing on nicotine replacement and non-nicotine replacement. Papers with titles and abstracts that used the term “drug” also used the terms “intervention”, “problem”, or “disorder”. The term “alcohol” frequently appeared in studies on adolescents.

### Effect

The term “effect” appeared 8640 times in the titles and abstracts, co-occurring with terms related to intervention/program or effects of “smoking”, “alcohol” and “drug” use on human health.

## Discussion

This study provided an insight of global publications trend, research productivity and collaboration networks in the field of substance use disorder and treatment. In this study, we demonstrated a rapid increase of publications in the past few decades, especially papers focusing on mindfulness and e-health interventions. This growth was led by leading institutions located in the United States of America, United Kingdom, Europe, Canada, and Australia.

The current finding suggested that the number of publications about traditional intervention approaches such as behavioral therapy, cognitive behavioral therapy, or counselling raised gradually over years. This result was in line with previous reviews that these methods were the most common way in intervening alcohol, tobacco and drug-use disorder [40–42]. Moreover, we found a substantial shift toward the use of mindfulness and e-health interventions in this field. In literature, mindfulness interventions were effective in preventing alcohol relapse and decreasing withdrawal symptoms when compared with cognitive-behavioral treatment [43, 44]. Also, publications about digital medicine (or e-health) were found in all three types of substance use disorder. This intervention is delivered via computers or mobile phones, which enables to facilitate interactions between patients and clinicians. Although its long-term effects were limited, digital medicine was found to be useful when combined with human supports [45].

We found a pattern of collaborative research networks that showed a preference for geographical proximity and cultural similarity. Northern European countries (Norway, Finland, and Sweden), three countries in The United Kingdom (England, Scotland, and Wales) created a cluster in collaboration in research. Meanwhile, another cluster among the U.S., Japan and China (mainland), Taiwan (China), Vietnam, South Africa, India and Ukraine was generated. This can be explained by the strong international scientific collaboration between US, Japan and Asia countries. Moreover, health topics were major concern of those developing countries and received support from developed countries [46, 47]. These findings suggest the importance of increasing research capacity and establish collaborative partnerships between high income and developing countries.

There are several research studies and policy related implications derived from our findings. First, our study found there was an upward trend in the employment of mindfulness or digital medicine in the treatment of substance use disorder. Mindfulness and digital medicine, although confirmed to be effective, they are only trial with small sample size and have not been evaluated in a long-term research. Therefore, more research are needed to evaluate the outcomes of two treatment for substance use disorder [9]. Second, other systematic-review and meta-analysis focused the treatment of only alcohol dependence, tobacco dependence or drug use disorder. Our research using bibliometric analysis, thus, we could identify the global trend of all three kinds of substance use disorders and also highlight gaps in the scientific literature regarding contextual factors and multi-level sociobiological. Third, our findings suggest the need for international policy efforts that place priority on the development of research capacity in settings where substance use disorder is prevalent, frequently where the availability of relevant resources is simultaneously low. Some of the heavily cited papers in our study dealt with implementation science and patient outcomes. We suggest the promotion of evidence-informed policy making, health system strengthening, a renewed focus on sociobiological causes of substance use disorder, and recommend the consideration of technological transfers as potential long and short-term measures. This suggests a need for research policy that supports the examination of interventions that culturally adhere to different local contexts, specifically those that place priority on the collective when addressing substance use disorder within their communities.

This is the first bibliometric analysis of substance use disorder treatment literature. The use of bibliometric and similar approaches, like scientometrics and informetrics, have been used to monitor the trends in other research areas. Bibliometric analyses serve as a helpful tool for research managers and policy makers when setting priorities and identifying strategies for research development and public resource allocation. Previous systematic reviews and meta-analyses of substance use disorder interventions have largely focused on specific issues and combined outcomes of different treatment options. Thus, the implications of these reviews were intended to inform clinical practice and the design of intervention programs. Meanwhile, our study provides an overarching view of the changes that have occurred within the substance use disorder research agenda over multiple decades. The study of these general trends is useful for clinicians, researchers, program managers, and policymakers having the over-trend of the global development in treatment for substance use disorder people. However, the application of those methods in a country different from one country to another, especially between developing countries and developed ones. Social, culture and environment can be the factors contributes to increase the differences in policy application. For instance, in Vietnam, alcohol is consumed in traditional national holidays such as the Lunar New Year, or weddings, housewarmings, even in funerals, and death anniversary, especially in mountainous or rural areas. Moreover, alcohol is familiar part of business transactions [48] . National legal minimum age for on- and off premise sales of alcohol is 18 and 21 in Vietnam and the U.S., respectively. Therefore, there is a need for increasing the perceptions of potential harms caused by alcohol and tighten the minimum age of alcohol using. Government should get the priorities in investing more research in substance use disorder research and treatment, alongside the need to understandings local contexts.

The findings of this study should be viewed in light of its limitations. First, as the scope of our search on drug use was limited to opioid, drug, substance and marijuana, publications on other specific substances like Amphetamine-type stimulants (ATS) or other stimulants may not be covered, which may impact the thoroughness of our results and analysis. The breadth and comprehensiveness of our study may also be influence by the restriction on types of publications included - which consisted of only searchable peer-reviewed research articles and reviews, as well as on language of publications - the selected documents for synthesis were written in English and work produced outside academic institutions that were written in local languages was excluded. As for keywords, our analysis of their occurrence and co-occurrence may not fully reflect the full content of the articles they are attached to [see Additional file 1]. However, as a bibliometric analysis of large volume of publications, a summary of keywords is a helpful proxy for the overall contents of these papers.

## Conclusions

Compared to other bibliometric analysis of substance use disorder, our bibliometric analysis offers a rare and comprehensive picture of the global efforts of substance use disorder and treatment. This study with the visualization of co-occurrence term in the titles and abstracts allows researchers to track connections among clusters, which is essential in identifying the global research trend. Researchers and policy makers can based on the results of this research to identify the future directions for research productions as well as the consider new therapy or prevention applying for the treatment of substance use disorder.

## Additional file

Additional file 1: **Figure S1.** Selection of papers. **Table S1.** Most prolific Institution/Organizations. **Table S2.** Most cited papers. **Figure S2.** Co-Occurrence of Author’s Keyword. (PDF 896 kb)

## Abbreviations

DALYs: Disability-adjusted life year
UNODC: United Nations Office on Drugs and Crime
WHO: World Health Organization
YLL: Years of life lost
