# Supplementary material for: Global research mapping of substance use disorder and treatment 1971–2017: implications for priority setting
Source: Subst Abuse Treat Prev Policy. 2019 May 17;14:21. doi: 10.1186/s13011-019-0204-7 (PMC6525403; doi:10.1186/s13011-019-0204-7)
Supplement: Supplementary file 1 — Figure S1. Selection of papers. Table S1. Most prolific Institution/Organizations. Table S2. Most cited papers. Figure S2. Co-Occurrence of Author’s Keyword. (PDF 896 kb) [file 13011_2019_204_MOESM1_ESM.pdf]

Additional file 1

Figure S1. Selection of papers.

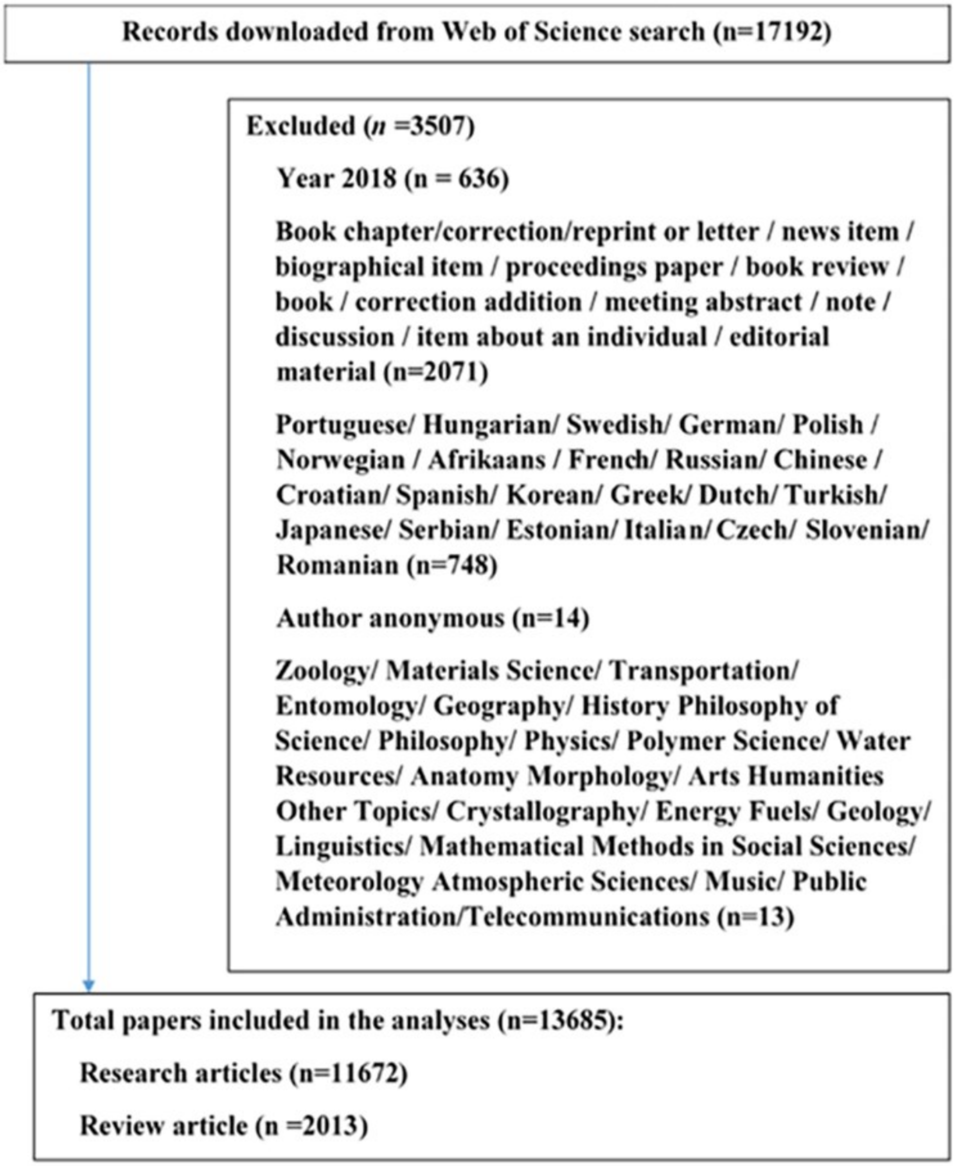

Figure S1 shows the stepwise search strategy utilized in this study. Our initial search on the WOS yielded 17,192 results published between 1971 and 2017. This was reduced by 3507 results after applying our inclusion and inclusion criteria. After this removal of unrelated data, our search yielded 13,685 substance abuse related research papers. Of these, 11,672 were original articles, and 2,013 were reviews.

Table S1. Most prolific Institution/Organizations

No	Institutions/organizations	Number	%	Country	Citation	Citations per paper	h-index
1	University of California System	896	6.5	United States	33696	37.6	89
2	Yale University	635	4.6	United States	23810	37.5	78
3	Harvard University	553	4.0	United States	20668	37.4	69
4	University of Pennsylvania	456	3.3	United States	18471	40.5	67
5	Johns Hopkins University	441	3.2	United States	15480	35.1	66
6	VA Boston Healthcare System	401	2.9	United States	15489	38.6	62
7	University of London	370	2.7	England	15550	42.0	61
8	Brown University	366	2.7	United States	13996	38.2	66
9	Columbia University	366	2.7	United States	14987	41.0	64
10	National Institutes of Health NIH	362	2.6	United States	15737	43.5	65
11	University of Texas System	360	2.6	United States	9692	26.9	51
12	Pennsylvania Commonwealth System of Higher Education	356	2.6	United States	13413	37.7	56
13	University of California Los Angeles	352	2.6	United States	12233	34.8	58
14	University of California San Francisco	340	2.5	United States	13781	40.5	63
15	University of Washington	311	2.3	United States	11348	36.5	52
16	University of Washington Seattle	307	2.2	United States	11314	36.9	52
17	University of Toronto	262	1.9	Canada	7448	28.4	45
18	State University System of Florida	236	1.7	United States	5514	23.4	37
19	US Department of Veteran Affairs	236	1.7	United States	7083	30.0	39
20	NIH National Institute on Drug Abuse	235	1.7	United States	9654	41.1	51
21	University System of Maryland	230	1.7	United States	7921	34.4	44
22	University of Pittsburgh	228	1.7	United States	8488	37.2	49
23	University of Minnesota System	221	1.6	United States	7710	34.9	45

24	University of Minnesota Twin Cities	220	1.6	United States	7702	35.0	45
25	Massachusetts General Hospital	204	1.5	United States	8857	43.4	48
26	Stanford University	202	1.5	United States	7839	38.8	50

The most prolific institutions are presented in **Table S1**. Institutions included in **Table S1** were required to have a minimum of 200 publications attributed to them. This attribution was determined based on the affiliation of the first author of each article. Among 26 universities/ organizations, 24 located in the United States. The University of California System was the most prolific entity within our dataset, scoring highest in terms of publication volume, number of total citations and their h-index. Massachusetts General Hospital had the highest number of citations per paper with 43.4. Among these institutions, only two were located outside of the United States. These included the University of London (United Kingdom) and the University of Toronto (Canada).

Table S2. Most cited papers

Rank	Paper	Total citation	Citations/ year
1	Hayes, SC; Luoma, JB; Bond, FW; Masuda, A; Lillis, J. Acceptance and commitment therapy: Model, processes and outcomes. Behavior Research and Therapy.2006;44:1-25	1482	134.7
2	Durlak, JA; DuPre, EP. Implementation matters: A review of research on the influence of implementation on program outcomes and the factors affecting implementation. American Journal of Community Psychology.2008;41:327-350	1345	149.4
3	Prince, M; Patel, V; Saxena, S; Maj, M; Maselko, J; Phillips, MR; Rahman, A. Global mental health 1 - No health without mental health. Lancet.2007;370:859-877	1245	124.5
4	Aldao, A; Nolen-Hoeksema, S; Schweizer, S. Emotion-regulation strategies across psychopathology: A meta-analytic review. Clinical Psychology Review.2010;30:217-237	1214	173.4
5	Chou, R; Fanciullo, GJ; Fine, PG; Adler, JA; Ballantyne, JC; Davies, P; Donovan, MI; Fishbain, DA; Foley, KM; Fudin, J; Gilson, AM; Kelter, A; Mauskop, A; O'Connor, PG; Passik, SD; Pasternak, GW; Portenoy, RK; Rich, BA; Roberts, RG; Todd, KH; Miaskowski, C.Clinical Guidelines for the Use of Chronic Opioid Therapy in Chronic Non-cancer Pain.Journal of Pain. 2009;10:113-130	1112	139.0
6	Jorenby, DE; Leischow, SJ; Nides, MA; Rennard, SI; Johnston, JA; Hughes, AR; Smith, SS; Muramoto, ML; Daughton, DM; Doan, K; Fiore, MC; Baker, TB. A controlled trial of sustained-release bupropion, a nicotine patch, or both for smoking cessation.New England Journal of Medicine.1999;340:685-691	1012	56.2
7	Jorenby, DE; Hays, JT; Rigotti, NA; Azoulay, S; Watsky, EJ; Williams, KE; Billing, CB; Gong, J; Reeves, KR.Efficacy of varenicline, an alpha 4 beta 2 nicotinic acetylcholine receptor partial agonist, vs placebo or sustained-release bupropion for smoking cessation - A randomized controlled trial. Jama- Jornal of The American Medical Association.2006;296:56-63	898	81.6
8	Gonzales, D; Rennard, SI; Nides, M; Oncken, C; Azoulay, S; Billing, CB; Watsky, EJ; Gong, J; Williams, KE; Reeves, KR. Varenicline, an alpha 4 beta 2 nicotinic acetylcholine receptor partial agonist, vs sustained-release bupropion and placebo for smoking cessation - A randomized controlled trial. Jama Journal of The American Medical Assciation.2006;296:47-55	884	80.4
9	Hurt, RD; Sachs, DPL; Glover, ED; Offord, KP; Johnston, JA; Dale, LC; Khayrallah, MA; Schroeder, DR; Glover, PN; Sullivan, CR; Croghan, IT; Sullivan, PM.A comparison of sustained-release bupropion and placebo for smoking cessation. New England Journal of Medicine. 1997;337:1195-1202	872	43.6
10	Noar, SM; Benac, CN; Harris, MS. Does tailoring matter? Meta-analytic review of tailored print health Behavior change interventions. Psychological Bullentin.2007;133:673-693	822	82.2

Rank	Paper	Total citation	Citations/ year
11	Rubak, S; Sandboek, A; Lauritzen, T; Christensen, B. Motivational interviewing: a systematic review and meta-analysis. British Journal of General Practice.2005;55:305-312	809	67.4
12	Burke, BL; Arkowitz, H; Menchola, M. The efficacy of motivational interviewing: A meta-analysis of controlled clinical trials. Journal of Consulting and Clinical Psychology.2003;71:843-861	796	56.9
13	Bickel, WK; Odum, AL; Madden, GJ. Impulsivity and cigarette smoking: delay discounting in current, never, and ex-smokers.Psychophrmacology.1999;146:447-454	730	40.6
14	Bond, C; LaForge, KS; Tian, MT; Melia, D; Zhang, SW; Borg, L; Gong, JH; Schluger, J; Strong, JA; Leal, SM; Tischfield, JA; Kreek, MJ; Yu, L. Single-nucleotide polymorphism in the human mu opioid receptor gene alters beta-endorphin binding and activity: Possible implications for opiate addiction. Proceedings of the National Academy of Sciences of The United States of America.1998;95:9608-9613	729	38.4
15	Hughes, JR; Keely, J; Naud, S. Shape of the relapse curve and long-term abstinence among untreated smokers.ADDICTION.2004;99:29-38	676	52.0
16	Stead, LF; Perera, R; Mant, D; Lancaster, T. Nicotine replacement therapy for smoking cessation (Review). Cochrane Database of Systematic Reviews.2008.	623	69.2
17	Putnam, FW. Ten-year research update review: Child sexual abuse. Journal of American Academy of Child and Adolescent Psychiatry.2003;42:269-278	621	44.4
18	Verdejo-Garcia, A; Lawrence, AJ; Clark, L. Impulsivity as a vulnerability marker for substance-use disorders: Review of findings from high-risk research, problem gamblers and genetic association studies. Neuroscience and Biobehavioral Reviews.2008;32:777-810	611	67.9
19	Chang, ET; Adami, HO.The enigmatic epidemiology of nasopharyngeal carcinoma. Cancer Epidemiology Biomarkers & Prevention.2006;15:1765-1777	606	55.1
20	Kendler, Ks; Neale, Mc; Maclean, Cj; Heath, Ac; Eaves, Lj; Kessler, Rc. Smoking and major depression - a causal-analysis. Archives of General Psychiatry.1993;50:36-43	600	25.0
21	Jeffery, RW; Drewnowski, A; Epstein, LH; Stunkard, AJ; Wilson, GT; Wing, RR; Hill, DR. Long-term maintenance of weight loss: Current status.Heath Psychology.2000;19:5-16	595	35.0
22	Mclellan, At; Arndt, Io; Metzger, Ds; Woody, Ge; Obrien, Cp. The Effects of Psychosocial Services In Substance-Abuse Treatment. JAMA-Journal of The American Medical Association. 1993;269:1953-1959	595	24.8
23	Coe, JW; Brooks, PR; Vetelino, MG; Wirtz, MC; Arnold, EP; Huang, JH; Sands, SB; Davis, TI; Lebel, LA; Fox, CB; Shrikhande, A; Heym, JH; Schaeffer, E; Rollema, H; Lu, Y; Mansbach, RS; Chambers, LK; Rovetti, CC; Schulz, DW; Tingley, FD; O'Neill, BT. Varenicline: An alpha 4 beta 2 nicotinic receptor partial agonist for smoking cessation. Journal of Medicinal Chemistry.2005;48:3474-3477	591	49.3
24	Ramsay, DJ; Bowman, MA; Greenman, PE; Jiang, SP; Kushi, LH; Leeman, S; Lin, KM; Moerman, DE; Schnoll, SH; Walker, M; Waternaux,	567	29.8

Rank	Paper	Total citation	Citations/ year
	C; Wisneski, LA. Acupuncture. JAMA Journal of The American Medical Association.1998;280:1518-1524		
25	Proctor, E; Silmere, H; Raghavan, R; Hovmand, P; Aarons, G; Bunger, A; Griffey, R; Hensley, M. Outcomes for Implementation Research: Conceptual Distinctions, Measurement Challenges, and Research Agenda. Administration and Policy in Mental Health and Mental Health Services Research.2011;38:65-76	546	91.0
26	West, R; McNeill, A; Raw, M. Smoking cessation guidelines for health professionals: an update.Thorax.2000;55:987-999	539	31.7
27	Miller, WR; Rose, GS. Toward a Theory of Motivational Interviewing. American Psychologist.2009;64:527-537	532	66.5
28	Fiore, MC; Bailey, WC; Cohen, SJ; Dorfman, SF; Fox, BJ; Goldstein, MG; Gritz, E; Hasselblad, V; Heyman, RB; Jaen, CR; Jorenby, D; Kottke, TE; Lando, HA; Mecklenburg, RE; Mullen, PD; Nett, L; Piper, M; Robinson, L; Stitzer, M; Tommasello, A; Welsch, S; Villejo, L; Wewers, ME; Baker, TB. A clinical practice guideline for treating tobacco use and dependence - A US Public Health Service report. Jama Journal of The American Medical Association.2000;283:3244-3254	521	30.6
29	Prochaska, Jo; Diclemente, Cc; Velicer, Wf; Rossi, Js. Standardized, Individualized, Interactive, and Personalized Self-help Programs for Smoking Cessation. Health Psychology.1993;12:399-405	521	21.7
30	Brooner, RK; King, VL; Kidorf, M; Schmidt, CW; Bigelow, GE. Psychiatric and substance use comorbidity among treatment-seeking opioid abusers.Archives of General Psychiatry.1997;54:71-80	511	25.6
31	Herz, A.Endogenous opioid systems and alcohol addiction. Psychopharmacology.1997;129:99-111	510	25.5
32	Dunn, C; Deroo, L; Rivara, FP. The use of brief interventions adapted from motivational interviewing across behavioral domains: a systematic review. Addiction.2001;96:1725-1742	506	31.6
33	Miller, WR; Yahne, CE; Moyers, TB; Martinez, J; Pirritano, M.A randomized trial of methods to help clinicians learn motivational interviewing. Journal of Consulting and Clinical Psychology.2004;72:1050-1062	500	38.5

Table S2 lists the top 33 papers with the highest number of citations. In total, all papers included in our dataset received 388,139 citations. Over 719 papers had more than 100 citations. Twenty-three journals published the top 33 papers included in our list. The most popular journals used by these high profiles papers included JAMA Journal of the American Medical Association, followed by Addiction, Archives of general psychiatry, Health Psychology, New England Journal of Medicine, Psychopharmacology, each of which contained two papers. **Table S2** also shows that these high profile papers were concerned with the following topics: **smoking and tobacco withdrawal** (paper number 1, 2, 6, 7, 8, 9,15,16,19, 20, 21, 23, 24, 26 and 29 in Table S2); **drug abuse** (paper number 4,13, 18, 22, 25, and 30 in Table 2); **alcohol addiction** (paper number 31, 32, and 33 in Table S2); **behavioral therapy** (paper number 1, 2, 3, 5 and 13 in Table S2); **nicotine replacement treatment** (paper number 6, 7, 9, 15, 16 and 28 in Table S2); **motivational interviewing** (paper number 11 and 27 in Table S2); **non-nicotine medication** (paper number

23 in Table S2); **acupuncture** (paper number 24 in Table S2); **methadone maintenance** (paper number 30 in Table S2); and **self-help** (paper number 29 in Table S2).

Figure S2. Co-Occurrence of Author's Keyword

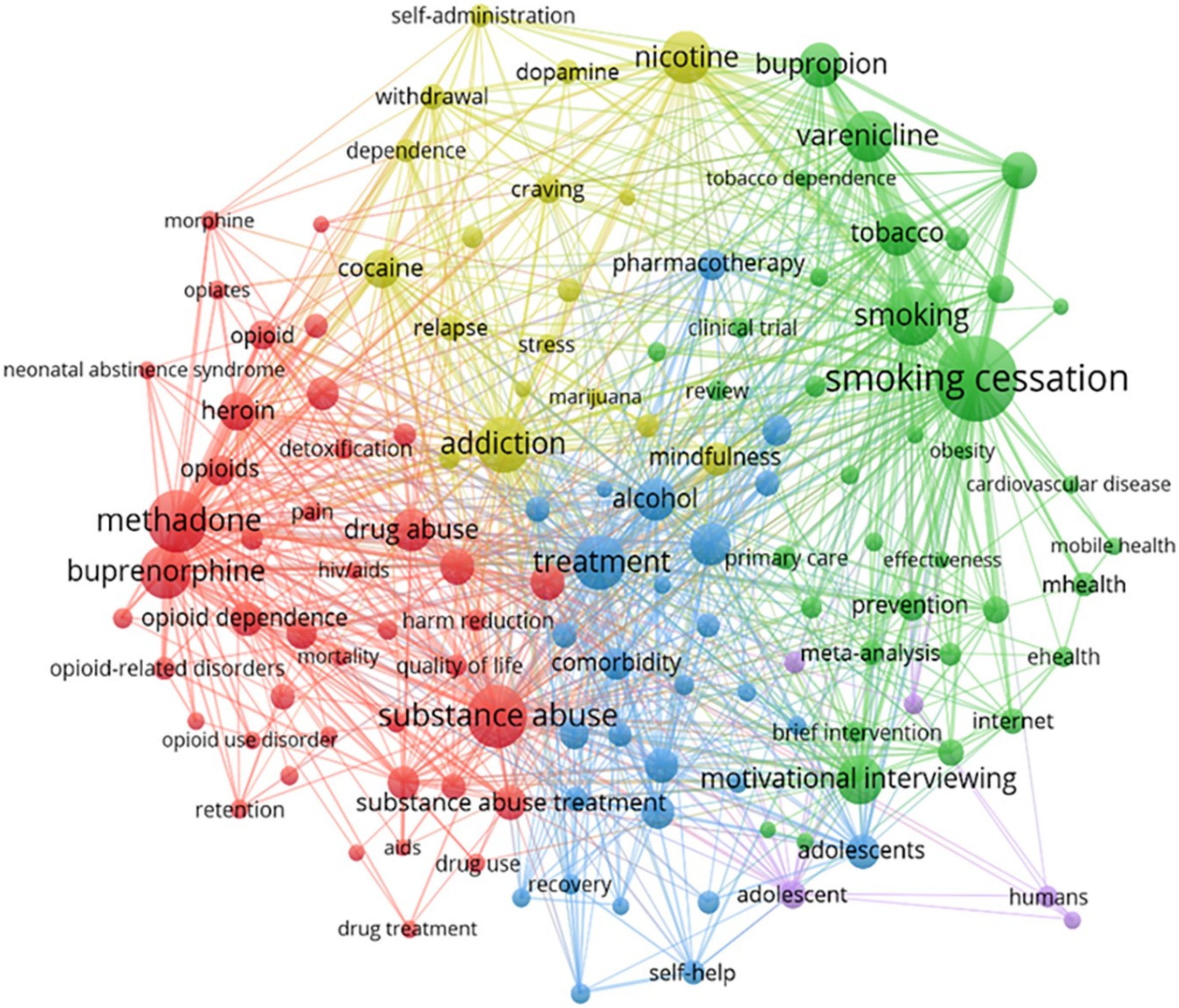

Figure S2 points out the keyword co-occurrence which is a measure of the probability that a particular word appears on the Web of Science search results. The thickness of lines is the strength of the relationship between keywords relative to the others. The strength of these relationships was determined by the frequency with which they appeared together in published articles. The position of a key word within this constellation represents how interrelated and frequent its co-occurrence was with other terms. There are 14,431 author keywords in the dataset. We applied the minimum cutoff of 50 times appearance with 129 keywords. These terms generally fell into 3 categories: 1) The type of substance abuse. The most common substance related keywords within our dataset were “Smoking” and “tobacco”. This was followed by “alcohol”, “cocaine” and “opioids”. Four major thematic categories emerged from our analysis of these clustered terms: a) Smoking Cessation (Blue). b) Substance Abuse (Green). c) Alcohol Abuse (Red) d) Pharmacotherapy Therapy (Yellow). 2) The consequences and comorbidity of substance abuse. For example, the terms “Substance abuse disorder”, “Depression”, “Anxiety” and “Schizophrenia” frequently occurred alongside “Alcohol”. Similarly, work exploring substance abuse frequently included keywords describing consequences such as “Addiction” and “Opioid Dependence”, alongside comorbid conditions such as “HIV”, “Pregnancy”, and “quality of life”. Within the “Smoking Cessation” theme, this work frequently used terms like “craving”, “withdraw” to label work focused on related consequences “obesity” or “cardiovascular”. 3) Interventions and therapy related to substance abuse. Within our “Smoking Cessation” theme, frequently co-occurring interventions included commonly used pharmacological interventions like “Varenicline” and “Bupropion”. In contrast, “Methadone Maintenance” and “Methadone” were the only key terms related to interventions within our “Substance Abuse” theme. The co-occurrence of intervention related keywords was most common within our Alcohol Abuse theme. While being related to many key terms within the entire figure, “Treatment” was a major node within the “Alcohol Abuse” constellation. This cluster of terms included alcohol related interventions, none of which were pharmacological. These included: “Prevention”, “Mindfulness” and “Motivational Interviewing”.
